# Gut Microbiota Profiling: Metabolomics Based Approach to Unravel Compounds Affecting Human Health

**DOI:** 10.3389/fmicb.2016.01144

**Published:** 2016-07-26

**Authors:** Pamela Vernocchi, Federica Del Chierico, Lorenza Putignani

**Affiliations:** ^1^Unit of Human Microbiome, Genetic and Rare Diseases Area, Bambino Gesù Children's Hospital, IRCCSRome, Italy; ^2^Unit of Parasitology, Bambino Gesù Children's Hospital, IRCCSRome, Italy

**Keywords:** gut microbiota, metabolome, state of health, diseases, dietary habits, omic approach

## Abstract

The gut microbiota is composed of a huge number of different bacteria, that produce a large amount of compounds playing a key role in microbe selection and in the construction of a metabolic signaling network. The microbial activities are affected by environmental *stimuli* leading to the generation of a wide number of compounds, that influence the host metabolome and human health. Indeed, metabolite profiles related to the gut microbiota can offer deep insights on the impact of lifestyle and dietary factors on chronic and acute diseases. Metagenomics, metaproteomics and metabolomics are some of the meta-omics approaches to study the modulation of the gut microbiota. Metabolomic research applied to biofluids allows to: define the metabolic profile; identify and quantify classes and compounds of interest; characterize small molecules produced by intestinal microbes; and define the biochemical pathways of metabolites. Mass spectrometry and nuclear magnetic resonance spectroscopy are the principal technologies applied to metabolomics in terms of coverage, sensitivity and quantification. Moreover, the use of biostatistics and mathematical approaches coupled with metabolomics play a key role in the extraction of biologically meaningful information from wide datasets. Metabolomic studies in gut microbiota-related research have increased, focusing on the generation of novel biomarkers, which could lead to the development of mechanistic hypotheses potentially applicable to the development of nutritional and personalized therapies.

## Introduction

The gut microbiota is exclusively responsible for several metabolic important functions, including vitamin and short chain fatty acid (SCFAs) production, amino acid (AAs) synthesis, bile acid biotransformation, hydrolysis and fermentation of non-digestible substrates (Putignani et al., 2015). The beneficial effects of gut microbiota include: (*i*) immune-cell homeostasis and development (Th1 vs. Th2 and Th17), (*ii*) epithelial homeostasis, (*iii*) enteric nerve regulation, (*iv*) support of angiogenesis, food digestion, and fat metabolism (Holmes et al., 2011).

The gut microbiota, through metabolite production/fermentation, modulates signaling pathways involved in the homeostasis of intestinal mucosa. When a balanced interaction between the gastrointestinal (GI) tract and the resident microbiota is disrupted, intestinal and extraintestinal diseases may develop (Putignani et al., 2015), such as allergy, inflammatory bowel disease (IBD), obesity, cancer and diabetes, metabolic disorders, cardiovascular dyslipidemia, and neuropathology (Holmes et al., 2011).

The advent of the omics-based systems biology era has opened a new scenario in the comprehension of the gut ecosystem by shedding light on its shape, modulation and interplay with microorganisms, food functionality, and the role of nutrients in health (Moco et al., 2013; Putignani et al., 2015).

The “omics” technologies are presently applied to: (*i*) determine specific disease markers and novel diagnostic targets; (*ii*) discover functional alterations in the physiopathology of several diseases; (*iii*) discover the relationship between the gut microbiota and the host metabolisms (De Preter and Verbeke, 2013). In particular, the use of metabolomics, being a well-established and powerful top–down systems biology approach, is crucial to unravel the genetics–environment–health relation, as well as the typical clinical biomarkers of the different diseases (Moco et al., 2013). In fact, metabolomics changed the concept that the cellular metabolism profile is complete (Dettmer et al., 2007). Therefore, metabolomics is useful to elucidate the complex interactions of components, to understand the whole system, and to discover new metabolites in order to provide both a different perspective on cellular homeostasis (Liu et al., 2010), and new, unexpected pathways which may have a key physiological role (Zamboni et al., 2015). The metabolomics experiment (sampling, sample preparation, instrumental analysis, data processing, and data interpretation) fulfill the goal of improving the current status of biological information associated to the metabolome and, more generally, to functional genomics (Harrigan and Goodacre, 2003). Nowadays, metabolomics is used to: (*i*) identify biomarkers that could indicate the presence of a diseases, or a response to drug intervention (Dunn and Ellis, 2005); (*ii*) determine biochemical or environmental stresses (Le Gall et al., 2003); (*iii*) characterize microbial metabolism (Vaidyanathan et al., 2002); and (*iv*) characterize human health or disease (Holmes et al., 2011).

**Figure 1:**
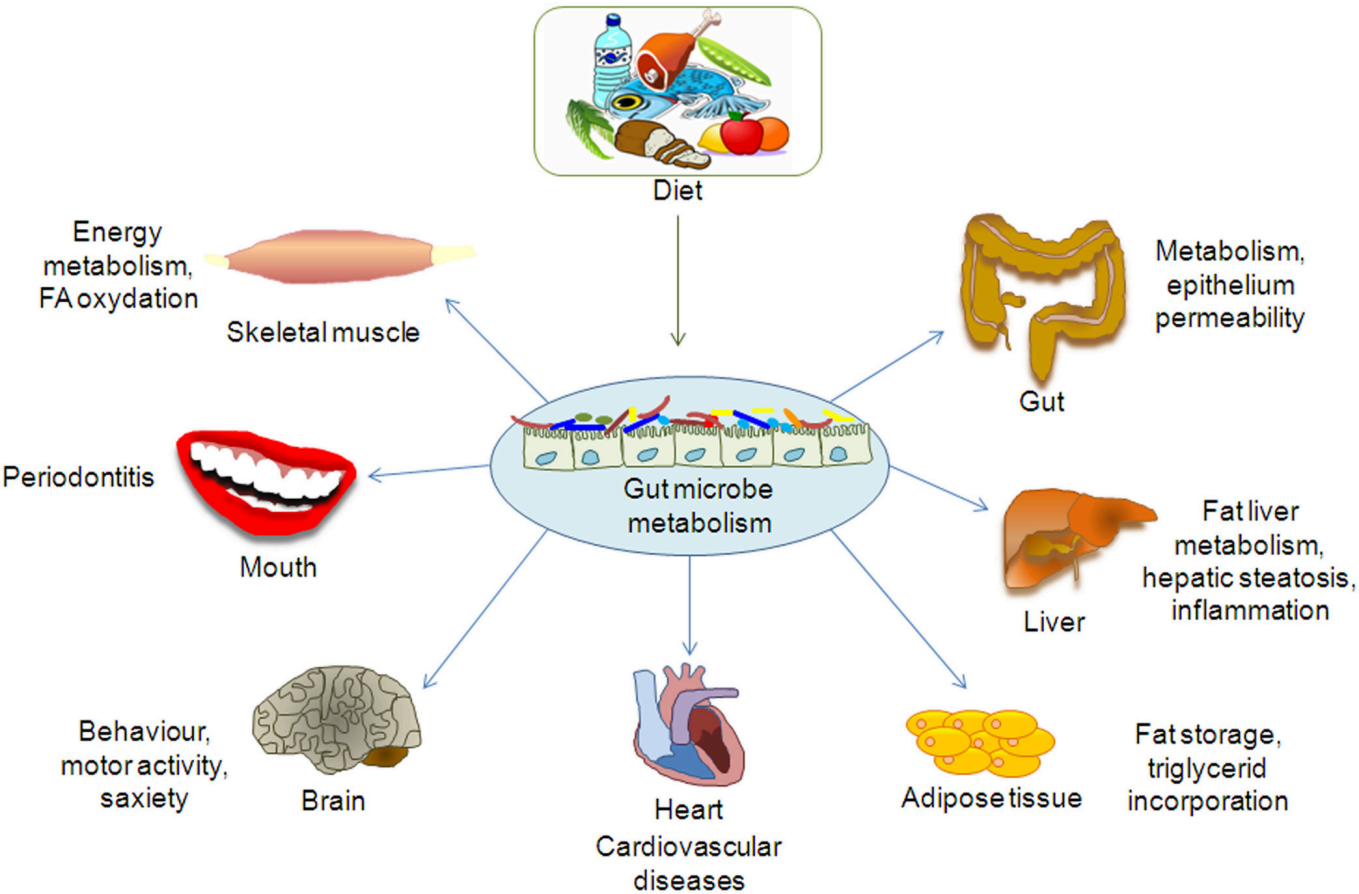
**Effect of gut microbiota metabolome on organs and tissues**.

Indeed, the metabolomics approach has been applied to several studies on the gut microbiota, mostly focused on the exploration of disease-related metabolites in order to obtain detailed information on the gut metabolic pathways. In fact, the gut microbiota is involved in several biochemical functions directly associated to the perturbation of specific gut microbial populations, which may lead to the development of diseases (De Preter, 2015; De Preter et al., 2015). In other words, as the gut microbiota interacts with the host metabolism and affects physiological or pathological conditions, (Figure 1; Table 1; Del Chierico et al., 2012) the study of its composition helps discriminate between unhealthy and healthy subjects.

**Table 1:** **Role of gut microbiota metabolites on health and disease**.

**Beneficial microbial activities**	**Benefits**	**References**	**Harmful microbial activities**	**Drawbacks**	**References**
SCFAs and vitamin production, recovery of energy	Nutrients and energy providing	Putignani et al., 2015	Lipopolysaccharide supply, inflammation	Obesity and metabolic syndrome	Krajmalnik-Brown et al., 2012
Butyrate production, fermentation of non-digestible fibers	Cancer prevention	Louis et al., 2014	Toxins production, inflammation	Cancer promotion	Louis et al., 2014
Antimicrobials production(e.g., bacteriocins, H_2_O_2_, acids etc.), intestinal pH regulation, competition for ecological niche	Inhibition of pathogens	Kamada et al., 2013	Tissue invasion, inflammation, disruption of the gut barrier/homeostasis	Infectious diseases, leaky gut	Kamada et al., 2013; Michielan and D'Incà, 2015
Anti-inflammatory vs. pro-inflammatory signals development	Normal gastrointestinal immune function	Belkaid and Hand, 2014	Pro-inflammatory vs. anti-inflammatory signals development	IBD, immune disorders	Putignani et al., 2015
Non-digestible carbohydrates metabolism	Normal gut motility	Flint et al., 2012	Metabolism imbalance	IBS, metabolic disease aggravation	Putignani et al., 2015
Propionate production	Gluconeogenesis, cholesterol synthesis inhibition	De Vadder et al., 2014	Acetate production	Cholesterol synthesis, cardiovascular diseases	Krajmalnik-Brown et al., 2012

Moreover, the identification of metabolites may highlight how lifestyle and dietary habits affect specific disease conditions (Vernocchi et al., 2012).

Finally, metabolomics represents an unprecedented approach to collect the complex metabolic interactions between the host and its commensal microbial partners, offering the opportunity to define individual and population phenotypes (Moco et al., 2013). In fact, several cellular metabolites are associated with the phenotypes of living organisms (i.e., human, mice, bacteria), and they represent the substrates and products of different biochemical pathways reflecting genetic and environmental factors (Kim et al., 2016).

Furthermore, these data will serve as a basis to comprehend, at the cellular and molecular levels, the relationships between nutritional status and disease risk predisposition, thus allowing to formulate nutritional recommendations.

This review is focused on the application of MS- and NMR-based metabolomic techniques to describe the gut microbiota metabolome and human physiology in relation to nutritional programs and therapies.

## Volatile and non-volatile compounds: detection methods and data analysis

Metabolomics uses high throughput techniques to characterize and quantify small molecules in several biofluids (urine, serum, plasma, feces, saliva), revealing a unique metabolic signature (Nicholson and Lindon, 2008).

However, due to the chemical diversity, the different properties of metabolites, and the large dynamic range of metabolite concentrations in samples, it is almost impossible to measure the complete metabolome with only one technique (De Preter and Verbeke, 2013; Smirnov et al., 2016). Considering that the amount of predictable metabolites and derivatives in mammals, plants and bacteria is unknown (Weckwerth and Morgenthal, 2005), there is the need of different analytical platforms and complex integrated computational pipelines, adjusted by analytical and chemical parameters, to cover complete metabolome pathways in the different biofluids (Savorani et al., 2013). Moreover, the collection and preparation of samples, and the selection of the appropriate analytical platforms are fundamental requisites for reproducibility of sample manipulation (Dunn and Ellis, 2005). Besides, the storage and the continuous sample freeze/thawing may alter the composition and stability of the samples and consequently the precision and accuracy of results (Roessner et al., 2000).

Finally, the fundamental requirements of metabolomics studies are: accurate study design; sample treatment and platform set up, corroborated by data analysis; integration of results; and biological interpretation (Smirnov et al., 2016).

Metabolomics can be divided into two different groups: targeted analysis and non-targeted discovery analysis (Dettmer et al., 2007). In particular, the targeted approach is related to the analysis of the different classes of molecules (i.e., carbohydrates, lipids, aminoacids), while non-targeted analysis gives a rapid snapshot of the metabolic profile of samples by using technologies able to detect a wide number of metabolites (Smirnov et al., 2016).

### Detection methods

At present, we are able to separate, detect, characterize, and quantify metabolites and their relevant metabolic pathways thanks to the rapid development of a range of analytical platforms, including gas chromatography (GC), liquid chromatography (LC), high pressure LC (HPLC), ultra pressure LC (UPLC), Fourier transform infrared spectroscopy (FTIR), ion cyclotrone resonance-FT (ICR-FT), capillary electrophoresis (CE) coupled to mass spectrometry (MS), and nuclear and proton nuclear magnetic resonance spectroscopy (NMR-^1^H-NMR) (Zheng et al., 2011; Vernocchi et al., 2012).

MS-based metabolomics allows targeted and untargeted metabolome analysis and it has become an indispensable tool in metabolome analysis (Milne et al., 2013); moreover, MS has a broader dynamic range, exhibits a high sensitivity and selectivity (Zhao and Hartung, 2015), and allows determining metabolite fingerprints for establishing metabolome libraries, which facilitate the identification of metabolites (Martins-de-Souza, 2014).

### Gas chromatography mass spectrometry

GC-MS is a combined system, with which thermally stable and volatile compounds are separated by GC and then eluting metabolites are detected by electron-impact (EI) mass spectrometers. GC-MS is considered as the gold standard in metabolomics (Harrigan and Goodacre, 2003). Even if GC has several advantages, such as high efficiency, reproducibility and sensitivity, it shows also some drawbacks. In fact, it can only be performed for volatile compounds, or those that can be made volatile, or made stable by derivatization (Roessner et al., 2000; Vernocchi et al., 2012).

Volatile organic compounds (VOCs) are important components of the metabolome (i.e., alcohols, esters, aldehydes, ketones, SCFAs) and are found in biological samples (Mills and Walker, 2001). The sample preparation methods consist of liquid or solid phase extraction (SPE) (Dettmer et al., 2007). Another rapid and solvent-free sample preparation technique is headspace-solid phase microextraction (HS-SPME) (Pawliszyn, 1997), for which the different types of stationary phases (polar and non-polar) used as fiber coatings are commercially available. On the other side, to stabilize the metabolites, two stages of derivatization with different kinds of reagents need to be performed (Roessner et al., 2000). During these processes, small aliquots of samples are analyzed by split or splitless mode on GC columns of various polarities, thus obtaining high chromatographic compound resolution and sensitivity, even if the resulted chromatograms are complex (i.e., multiple derivatization products), contain many metabolite peaks, and need a long run time (longer than 60 min) (Roessner et al., 2000). Therefore, coupling GC with time-of-flight (TOF)-MS, which has high scan rates and produces accurate peak deconvolution of complex samples in faster times, allows improving conventional GC–MS techniques in the analysis of ultra-complex samples (Dunn and Ellis, 2005; Dettmer et al., 2007).

Finally, metabolite quantification is obtained by external calibration or response ratio (peak area of metabolite/peak area of internal standard), while metabolite identification is obtained by matching retention time and mass spectrum of the sample peak with a pure compound previously analyzed, under identical instrumental conditions, with the same or different instruments (Fiehn et al., 2000), or by matching the metabolite against commercial databases (i.e., NIST, WILEY, EPA, NIH).

GC-MS can be used in several fields, such as plant metabolomics, as reported by Stashenko et al. (2004), who used SPME-GC-MS for sampling the volatile plant metabolites, or for example, by Akhatou et al. (2016), who combined GC-MS with multivariate statistical techniques to characterize the primary metabolome of different strawberry cultivars, and to study the influence of multiple agronomic conditions. Moreover, as reported by Currie et al. (2016), GC-MS has proved useful in microbial metabolomics related to pharmaceutical studies to analyze the endogenous metabolite levels produced by *Pseudomonas putida* in response to six pharmaceuticals; or in food studies, when used to characterize the microbial metabolite production in: cheese (Vannini et al., 2008; Pisano et al., 2016), probiotic food (Patrignani et al., 2009; Tabanelli et al., 2015b), sourdough (Guerzoni et al., 2007), wine (Vernocchi et al., 2011; Patrignani et al., 2016), sausages (Tabanelli et al., 2015a), and ready to eat products (Siroli et al., 2015). Moreover, GC-MS is used in clinical applications, for example to analyze volatile compounds (SPME-GC-MS) in urine, blood, feces, hair, breath and saliva (Mills and Walker, 2001), or to evaluate biomarkers in several diseases, such as asthma (Gahleitner et al., 2013; Chang et al., 2015), schizophrenia (Liu et al., 2010), depressive disorders (Ding et al., 2014), ulcerative colitis (Kohashi et al., 2014), and neonatal sepsis (Fanos et al., 2014). In the last years, GC-MS has become one of the most used techniques to study the modulation of gut microbiota as a result of nutrition (i.e., diet, nutraceutical food consumption), diseases, drug, and probiotic administration. Garner et al. (2007) qualitatively and quantitatively analyzed the fecal metabolome to identify potential biomarkers in GI diseases; Di Cagno et al. (2011) characterized the fecal metabolome of celiac children subjected to gluten-free diet, compared to healthy children; Francavilla et al. (2012) evaluated the gut metabolome of allergic children; Vitali et al. analyzed the effects of symbiotic or prebiotic foods and probiotic foods on the human gut metabolome profile (Vitali et al., 2010, 2012); De Filippis et al. evaluated the effects of the Mediterranean diet on the gut microbiota metabolome (De Filippis et al., 2015).

Moreover, De Preter (2015) and De Preter et al. (2015) applied this technique to make a clinical diagnosis of IBD, and to determine the impact of prebiotics on Chron's disease. Del Chierico et al. characterized the gut microbiota of non-alcoholic fatty liver disease (NAFLD), and obese pediatric patients to unravel disease signatures (Del Chierico et al., 2016). Finally, De Angelis et al. analyzed the fecal metabolome of children with autism and pervasive developmental disorders (De Angelis et al., 2013).

### Liquid chromatography mass spectrometry

HPLC separation may cover a wide range of compounds' determination, even though its resolution is low. LC is probably the most flexible separation method, as it allows to separate compounds with little effort in few pre-analytical steps (compared to GC-MS) (Moco et al., 2007). Usually, the metabolite separation obtained with LC is followed by electrospray ionization (ESI) or, to a lesser extent, by atmospheric pressure chemical ionization (APCI) (Bakhtiar et al., 2004). The combination of LC with MS allows to analyze polar, non-polar and neutral compounds separately in a complex matrix (Smirnov et al., 2016). This technique diverges from GC-MS for the lower temperatures of analysis, and it does not require sample volatility, thus entailing an easier sample preparation (Dunn and Ellis, 2005).

LC/MS is an excellent technique showing sensitivity, specificity, resolving power, and capability to extract additional information about metabolites from their retention time (RT) domain (Forcisi et al., 2015).

Sample derivatization is commonly not necessary, although it can be helpful to improve the chromatographic sensitivity and resolution (Leavens et al., 2002), or to produce ionisable groups of metabolites otherwise not detectable by electrospray ionization (ESI) MS. Metabolite quantification is obtained by external calibration or response ratio, and metabolite identification is more time intensive. Moreover, ESI does not produce molecular ion fragmentation as it occurs by electron impact MS, so it does not provide direct metabolite identification by ESI mass spectra comparison, as ESI mass spectral libraries are not generally available. Nevertheless, accurate mass measurements can be obtained by coupling MS/MS using metabolite identification (Lenz et al., 2004). The advent of HPLC and UPLC allowed to shorten the analyzing time, provided higher resolution, sensitivity and efficiency, and permitted to reduce the quantity of samples and solvent necessary for the analysis (Smirnov et al., 2016).

The application of LC/MS allows the identification of target metabolites within a complex sample, not only with the information about monoisotopic mass, but also providing advice on the metabolite structure (Villas-Bôas et al., 2005).

LC-MS applications mainly concern the clinical and pharmaceutical fields (Bakhtiar et al., 2004). Nardotto et al. (2016) used LC/MS/MS systems to investigate patients with type 2 diabetes mellitus treated with an oral dose of racemic carvedilol, who showed accumulation in plasma. Mueller et al. applied this technique to measure plasma concentrations of trimethylamine-N-oxide, betaine and choline in the evaluation of patients with suspected coronary artery disease (Mueller et al., 2015).

An example of LC and GC technologies' combination, is also given by Chow et al. who studied the fecal metabolome, including the application of non-targeted metabolomics to separate breast-fed from formula-fed infants by using GC/MS and LC/MS/MS analysis to identify the various metabolites undergoing change (Chow et al., 2014).

### Capillary electrophoresis mass spectrometry

CE may offer high-analyte resolution and detect a wider spectrum of (polar) compounds compared to HPLC, but it is properly applicable only to charged analytes (Ramautar et al., 2013). However, only a few studies on this subject have been published to date, such as Soga et al. (2003), who separated cationic, anionic nucleotidis, and CoA metabolites to describe the coverage of the metabolome. These authors analyzed 1692 metabolites in bacterial extract (Chow et al., 2014).

### Fourier transform infrared spectroscopy

FT-IR spectroscopy allows rapid, non-destructive and high-throughput determination of different sample types. In particular, it can simultaneously detect different molecules, such as lipids and fatty acids (FAs), proteins, peptides, carbohydrates, polysaccharides, nucleic acids, (Harrigan and Goodacre, 2003; Dole et al., 2011), but sensitivity and selectivity of this technique are not high. On the contrary, ICR-FT/MS offers an ultrahigh mass resolution able to distinguish slight variations in a wide number of mass signals (Rosselló-Mora et al., 2008), and allowing to obtain the structural identification of new biomarkers (Jansson et al., 2009). In fact, Jansson et al. (2009) used ICR-FT/MS to distinguish between the masses of fecal metabolites in Chron's disease patients and healthy subjects.

Furthermore, FT-IR is principally useful for the identification of functional groups (Vernocchi et al., 2012). In fact, FT-IR has been used to assist infrared imaging in the diagnosis of many diseases, such as Parkinson, cancer, Alzheimer, kidney stone, arthritis (Dole et al., 2011), diabetes, and early stage insulin resistance (Chen et al., 2008).

Moreover, it is possible to combine the LC and FT-IR techniques, as performed by Walker et al. (2014), who identified taurine and sulfate conjugated fatty acids in feces of diabetic mice by coupling ICR-FT/MS and UPLC-MS.

Finally, MS-based metabolomic techniques offer high selectivity and sensitivity for metabolites' identification and quantification. In fact, they are considered as the most appropriate techniques for the detection of large numbers of metabolites, and, in combination with advanced and high-throughput platforms, they may help decrease the complexity of metabolite separation (Zheng et al., 2011; Zhao and Hartung, 2015). In particular, for the total screening of the small molecules in a biological system, MS non-targeted metabolomics is a powerful tool for the identification of metabolite signals present in spectra (Naz et al., 2014). The identification can be partly completed by matching against metabolite and spectral databases, such as METLIN (Smith et al., 2005), HMDB (Wishart et al., 2013), or ChemSpider (Pence and Williams, 2010). To describe the metabolic pathway of biological systems, it is also possible to refer to databases such as KEGG (Kanehisa et al., 2007), or Meta-Cyc (Caspi et al., 2007).

### Nuclear magnetic resonance spectroscopy

NMR spectroscopy, instead, uses the intramolecular magnetic field around atoms in molecules to change the resonance frequency, thus allowing access to details of molecules' electronic structure and obtaining information about their dynamics, reaction state, and chemical environment. Moreover, a minimal sample preparation is necessary for biofluids, except for feces or gut luminal content, which require the removal of undigested material, dead microbes and other particles (Smirnov et al., 2016). For feces in particular, samples are prepared using methanol (for lipophilic compounds as lipids, cholate, small phenolic acids) or water (for aminoacids, glucose, glycerol) (Jacobs et al., 2008; Lamichhane et al., 2015).

NMR spectroscopy is mainly useful to determine metabolic fingerprints leading to the identification and quantification of compounds in a non-targeted large-scale, in a non-destructive way, and with a high reproducibility (Lenz et al., 2004; Smolinska et al., 2012).

However, it is a relatively insensitive technique, and can only detect metabolites in high concentrations. The annotation is restricted to a limited number of low molecular weight molecules, and this is the major pitfall of this application (Jansson et al., 2009; Zhao and Hartung, 2015). Indeed, sensitivity depends on the natural concentration of the atoms in the matrix and, to improve sensitivity, long times of analysis, higher magnetic fields, and cryogenic probes are needed (Keun et al., 2002).

Another type of NMR spectroscopy is ^1^H (proton) NMR, which is unbiased to particular metabolites (Dunn and Ellis, 2005), unlike the other techniques discussed above. The NMR spectrum (chemical shift) depends on the shielding from electrons orbiting the nucleus, whereas for ^1^H-NMR the chemical shift is arranged as the difference between the resonance frequency of the observed proton and that of a reference proton in a reference metabolite (tetramethylsilane in solution, set at 0 ppm) (Dunn and Ellis, 2005).

The obtained spectra are complex and contain a wide number of signals, and frequently pure metabolites can be added to give a more in-depth clarification. This technique is frequently employed in clinical and pharmaceutical research and applications, in particular in the analysis of biofluids and tissues, where ^1^H-NMR is used to detect the modulation of metabolites in response to cellular stresses (Lindon et al., 2003). This has also been reported by Bro et al. (2015), who used plasma to determine breast cancer biomarkers, or by Villaseñor et al. (2014), who described the global metabolic phenotyping of acute pancreatitis, and by Dumas et al. (2016), who used this technique to study metabolic syndrome and fatty liver disease.

Moreover, when there is the need to understand how the diet or other external *stimuli* or diseases affect the microbiome composition (i.e., gut, urine, salive), metabolite detection is performed using this functional technique. In fact, several research studies have been conducted using NMR and ^1^H-NMR, for instance: Ndagijimana et al. (2009) described the effects of symbiotic food on human gut metabolic profile; Martin et al. (2012) studied the influence of gut metabolome on health and diseases; Bjerrum et al. (2015) investigated the gut metabolic biomarkers characterizing Chron's disease, ulcerative colitis and healthy subjects; Zhang et al. (2015) studied how the gut microbiota metabolome could alleviate obesity in children; Laghi et al. (2014) studied the antibiotic effect on vaginal microbiome; and Holmes et al. (2008) analyzed urine samples to discriminate metabolites across populations in order to identify major risk factors for coronary heart disease and stroke.

Hence, MS and ^1^H-NMR are by far the most frequently applied and the most powerful techniques in metabolomics (Collino et al., 2012). Recent advances in NMR and MS have allowed to evaluate at the same time thousands of metabolites related to the “metabolome,” and to define the end-points of metabolic processes in living systems (Nicholson et al., 2005).

In particular, ^1^H-NMR is currently the most used analytical technique for metabolite profiling compared to MS, while the combination of ^1^H-NMR and MS technologies would result in a better coverage of the complete metabolome (Bjerrum et al., 2015; De Preter, 2015; Wissenbach et al., 2016). In fact, by coupling metabolite separation technologies with spectrometry and spectroscopy, it is possible to reach a multidimensional approach leading to the structural identification of new metabolites (Chen et al., 2008).

**Figure 2:**
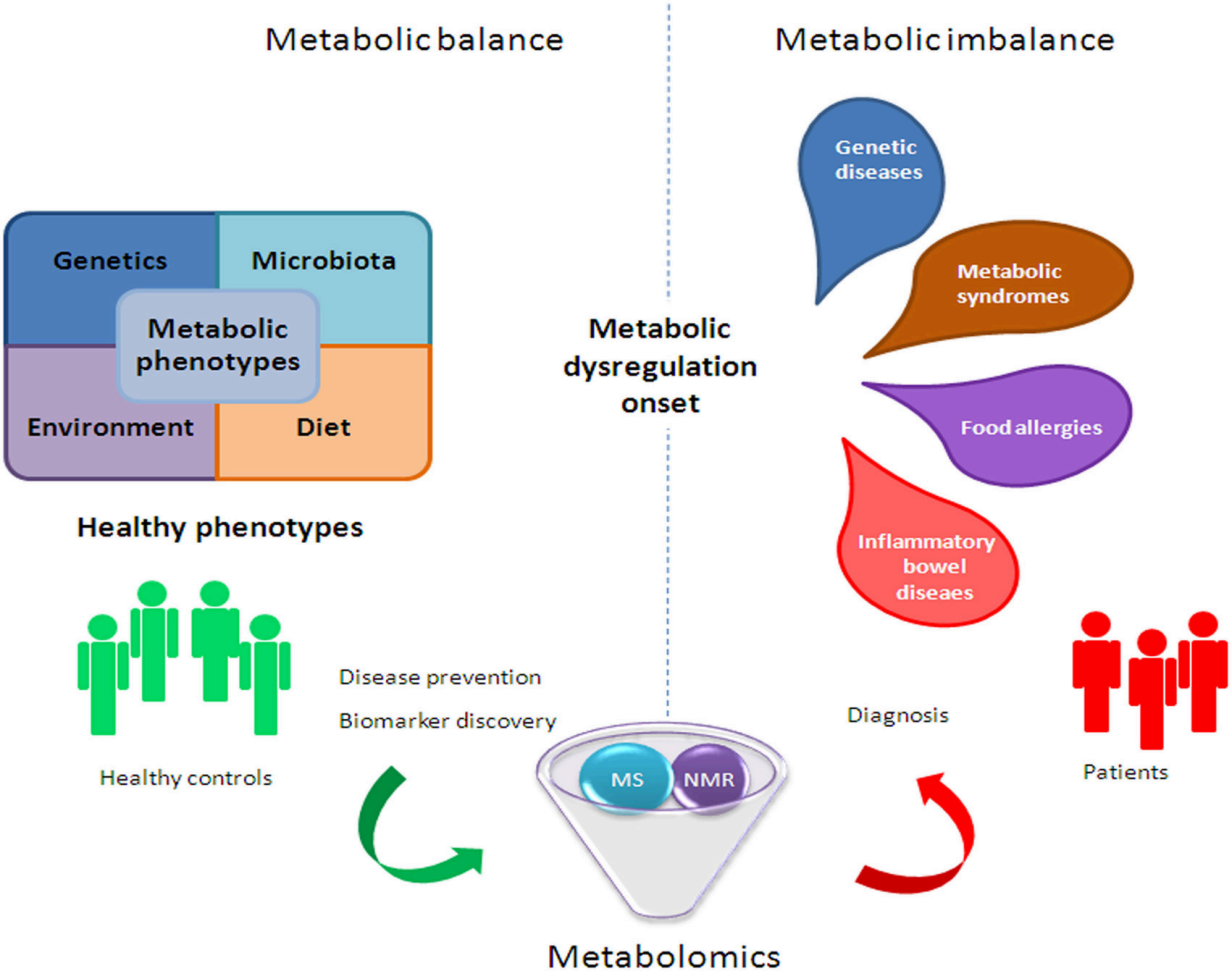
**Metabolomic application in human health to improve clinical and nutritional settings**.

However, the challenge for metabolomics is not only to discover unknown chemical structures, but also to generate meta-information, (i.e., sample origin, tissue, experimental conditions) in an accessible format (Weckwerth and Morgenthal, 2005). Thus, the structural identification of metabolites as potential biomarkers associated with diseases will be a major task of biological interpretation (Nassar and Talaat, 2004). In fact, small molecule metabolites are able to provide new mechanistic information on novel disease biomarkers, which is extremely important, given the paucity of existing markers. Moreover, metabolomics can induce significant progress in the identification of metabolomic fingerprints (which could be used as crucial diagnostic biomarkers) by producing a comprehensive map of metabolic pathway regulations, which represent the downstream expression of genome, transcriptome, and proteome. This comprehensive map may help define the phenotype of an organism at a specific time (Zhang et al., 2012). Therefore, the analysis of metabolic differences between unperturbed and perturbed pathways could provide insights on the underlying disease prognosis and diagnosis (Zhang et al., 2012; Figure 2).

### Data analysis

Hence, statistic and bioinformatic techniques are used for data mining complex metabolic profiles containing information related to genetics, environmental factors, gut microbiota activity, lifestyle, and eating habits. These strategies support the complicated process of identifying new biomarkers, which could indicate the individual response to specific physiological factors and/or nutritional interventions, and manage the relevant biological outcomes (Moco et al., 2013).

The application of biostatistics and mathematical approach has a key role in the extraction of biologically meaningful information from wide datasets. In computational analysis, the problems derive from a small batch of samples in contrast with the high number of detected metabolites, and in the consequent high dimensionality of the data matrix (Weckwerth and Morgenthal, 2005). Therefore, different statistical tools can be employed to discriminate among the samples and within the sample set (Worley and Powers, 2012).

In particular, diverse univariate and multivariate methods can be used as parametric (i.e., Student *t*-test, multivariate linear regression) or non-parametric (i.e., Mann-Whitney, Kruskal-Wallis) tests. Moreover, these methods can be divided into unsupervised techniques (i.e., principal component analysis (PCA), hierarchical cluster analysis), and supervised techniques (i.e., linear discriminant analysis, k-nearest neighbor). Among the supervised multivariate techniques, the partial least squares discriminant analysis (PLS-DA) has proved to be a particularly useful tool, in the presence of an irresolute sample separation obtained from PCA as it offers the possibility to use *a priori* information based on replicates (Raamsdonk et al., 2001; Gromski et al., 2015). PLS-DA is also used for its ability to identify potential biomarkers.

Moreover, statistical analyses of multivariate datasets allow to visualize the biological and molecular consistency. This consistency is based on the correlated functioning of metabolites in response to external conditions. Finally, correlation networks represent fingerprints of biochemical interactions, like the regulation of enzyme activity, and the interplay of anabolism and catabolism between the host and gut microbes (Weckwerth and Morgenthal, 2005).

## Metabolites associated with microbial metabolism or microbial–host cometabolism

The gut microbiota operates in a combined way with the host through the metabolic communication enacted by the different bacterial genera and species responsible for metabolite production (Table 2).

**Table 2:** **Metabolites associated with microbial metabolism or microbial–host cometabolism**.

**Metabolites**	**Bacteria**	**Biological functions**	**References**
**BACTERIAL METABOLISM**			
*SCFAs: acetate, propionate, butyrate;* branched CFAs: iso-butyrate, valerate and iso-valerate	Clostridial clusters IV and XIVa *Lactobacillus, Eubacterium, Roseburia, Faecalibacterium, Coprococcus*	Increasing cholesterol synthesis (acetate); gluconeogenesis (propionate); energy source for colonocytes (butyrate); cholesterol synthesis inhibition; linked to: cardiovascular disease, ulcerative colitis, Crohn's disease, antibiotic-associated diarrhea, obesity, metabolic syndrome, bowel disorders and cancer	Harig et al., 1989; Scheppach et al., 1991; Scheppach, 1994; Sabatino et al., 2005; Binder, 2010; Donohoe et al., 2011; Fukuda et al., 2011; Nicholson et al., 2012; Chambers et al., 2015
*Organic acids:* benzoate, hippurate, phenylacetate, phenylpropionate, hydroxybenzoate, hydroxyphenylacetate, hydroxyphenylpropionate 3,4-dihydroxyphenylpropionat and D-lactate	*Clostridium difficile, Faecalibacterium prausnitzii, Bifidobacterium, Subdoligranulum, Lactobacillus*	Related to hypertension and obesity, colorectal cancer, autism in children in humans and diabetes in a rat model	Lord and Bralley, 2008; Calvani et al., 2010; Qiu et al., 2010; Zhao et al., 2010; Zheng et al., 2011; Nicholson et al., 2012
*Vitamins:* vitamin B9, vitamin B2, vitamin B12, niacin, pyridoxine, vitamin K, vitamin B1, vitamin B5, vitamin B8	*Bifidobacterium bifidum, Bifidobacterium longum* subsp. *infantis, Bifidobacterium breve, B. longum* subsp. *longum Bifidobacterium adolescentis*, commensal Lactobacilli, *Bacillus subtilis Escherichia coli* and anaerobes, Bacteroidetes, Fusobacteria, Proteobacteria, Actinobacteria	Cellular metabolism	Deguchi et al., 1985; Noda et al., 1994; Roth et al., 1996; Bacher et al., 2000; Perkins and Pero, 2002; Stanton et al., 2005; Pompei et al., 2007; Smith et al., 2007; Rossi and Amaretti, 2010; Magnúúsdóóttir, et al., 2015
**BACTERIAL TRANSFORMED COMPOUNDS**			
*Bile salts:* cholate, hyocholate, deoxycholate, chenodeoxycholate, α-muricholate, β-muricholate, ω-muricholate, taurocholate, glycocholate, taurochenoxycholate, glycochenodeoxycholate, taurocholate, tauro–α–muricholate, tauro–β–muricholate, lithocholate, ursodeoxycholate, hyodeoxycholate, glycodeoxylcholate, taurohyocholate, taurodeoxylcholate	*Bacteroides, Clostridium, Lactobacillus, Bifidobacterium, Enterobacter, Eubacterium, Escherichia*	Absorption of dietary fats and lipid-soluble vitamins, facilitate lipid assimilation, maintain gut barrier function, regulate triglycerides, cholesterol and glucose by endocrine functions and energy homeostasis. Secondary bile salts linked to colon cancer.	Lis et al., 1976; Russell and Setchell, 1992; Groh et al., 1993; Ridlon et al., 2006; Dawson et al., 2009; Suhre et al., 2010; Nicholson et al., 2012
*Polyphenol:* Hydroxycinnamic acids and flavonoids	*Lactobacillus, Bifidobacterium*	Secondary metabolites production	Couteau et al., 2001; Clifford, 2004; Manach et al., 2004; Taverniti and Guglielmetti, 2012; Amaretti et al., 2015; Marín et al., 2015; Raimondi et al., 2015
*Lipids:* glycerol	*Bifidobacterium, Roseburia, Lactobacillus, Klebsiella, Enterobacter, Citrobacter, Clostridium*	Intestinal permeability, glucose homeostasis, promotion of chronic systemic inflammation by LPS; hyperinsulinemia improvement by conjugated FAs, immune system enhancement and lipoprotein profiles alteration.	Holmes et al., 2011; Nicholson et al., 2012
*Amino Acids*	Colonic bacteria*, Clostridium, Peptostreptococcus anaerobius*	ammonia production by deamination, amines production by decarboxylation	Moss et al., 1970; Clinton et al., 1988; Macfarlane and Macfarlane, 1995

### Bacterial metabolism

#### SCFAs

Several substances present in the large intestine, including indigestible oligosaccharides, dietary plant polysaccharides or fibers, non-digested proteins and intestinal mucin are fermented by the microbiota populations to produce SCFAs (Arora and Sharma, 2011). In particular, clostridial clusters IV, XIVa (e.g., *Eubacterium, Roseburia, Faecalibacterium*, and *Coprococcus* spp.) and *Lactobacillus* belonging to the phylum Firmicutes and the groups of Actinobacteria (*Bifidobacterium* spp.) are the main bacteria playing a central role in SCFAs metabolism (Nicholson et al., 2012; van Zanten et al., 2014).

Acetate is an important SCFA present in the colon, which could have a trophic effect on the colonic epithelium not only by local action, but also by raising the mucosal blood flux. Moreover, after transport to the portal circulation across the colonic mucosa, acetate passes through the liver and is regained in peripheral blood. Acetate's effect at the ileal level exceeds that of mixed SCFA (Scheppach, 1994). However, acetate is adsorbed by tissues involved in the rise of cholesterol synthesis (Scheppach et al., 1991). On the other side, propionate inhibits cholesterol synthesis (Scheppach, 1994; Wong et al., 2006). In fact, substrates that can decrease the acetate/propionate ratio may diminish serum lipids and consequently decrease the risk of cardiovascular disease (Wong et al., 2006). Butyrate represents the major energy source for colonocytes and has been studied for its role in nourishing the colonic mucosa and preventing colon cancer by promoting cell differentiation, cell-cycle arrest and apoptosis of transformed colonocytes (Scheppach, 1994; Velázquez et al., 1997; Walton et al., 2013).

Furthermore, butyrate improves insulin sensitivity and raises energy consumption in obese mice submitted to dietary regimen (Gao et al., 2009), and butyrate irrigation (enema) improves inflammation in diversion colitis (Scheppach et al., 1992; Wong et al., 2006). Butyrate and propionate, but not acetate, induce the production of gut hormones and reduce food intake (Lin et al., 2012). The treatment with acetate induces a marked reduction in lipid accumulation in the adipose tissue, protects against accumulation of fat in the liver, and improves glucose tolerance (Yamashita et al., 2007). In obese subjects, propionate significantly increases the release of post-prandial plasma peptide YY and glucagon-like peptide-1 from colonic cells, and reduces energy intake (Chambers et al., 2015). Chambers et al. (2015) found that inulin-propionate ester administrated to overweight adults significantly reduced weight gain, intra-abdominal adipose tissue distribution, and intrahepatocellular lipid content, and improved insulin resistance in the inulin control group.

Furthermore, other clinical studies demonstrated that the administration of SCFAs has a positive effect on the treatment of ulcerative colitis, Crohn's disease, and antibiotic-associated diarrhea and obesity, metabolic syndrome, bowel disorders, and cancer (Harig et al., 1989; Sabatino et al., 2005; Binder, 2010; Donohoe et al., 2011; Fukuda et al., 2011; Chambers et al., 2015). The degradation of proteins and amino acids by gut microbes also forms small amounts of branched chain FAs (iso-butyrate, valerate and iso-valerate) (Macfarlane and Gibson, 2004). SCFAs can be detected by using both the GC-MS and ^1^H-NMR spectroscopy techniques.

#### Organic acids

Several organic acids result from bacterial metabolism of dietary polyphenols or unassimilated AAs or carbohydrates (Lord and Bralley, 2008). High levels of organic acids in urines are associated with microbial overgrowth (Lord and Bralley, 2008). In particular, the hyperproduction of organic acids is associated with the overgrowth of *Clostridium difficile, Faecalibacterium prausnitzii, Bifidobacterium* spp., *Subdoligranulum* spp., *Lactobacillus* (Lord and Bralley, 2008; Nicholson et al., 2012).

Amongst organic acids, urinary hippuric acid may be a biomarker of hypertension and obesity in humans, while urinary 4-hydroxyphenylacetate and phenylacetate are potential biomarkers of colorectal cancer (Nicholson et al., 2012).

Lactic acid is the main product in the lactic acid bacteria (LAB) fermentation process. LAB are Gram+ and constitute a heterogeneous group of microorganisms that can also produce proteinaceous antimicrobial molecules, known as bacteriocins, that can help the producer microorganism to outcompete other bacterial species (Alvarez-Sieiro et al., 2016). Moreover, lactic acid represents a secondary metabolite that can be converted by clostridial cluster XIVa species into butyrate, or by clostridial cluster IX into propionate (Louis et al., 2007), thus inducing benefits by inhibiting both the propagation of harmful bacteria, and the production of putrefactive intestinal products. Lactic acid also participates in the intestinal peristalsis regulation (Sugawara et al., 2016). Furthermore, lactic acid is correlated to healthy vaginal microbiota, in fact it decreases in bacterial vaginosis, and it's produced by microbial species such as *Lactobacillus crispatus* and *Lactobacillus jensenii* (Vitali et al., 2007, 2015; Cruciani et al., 2015; Srinivasan et al., 2015). The detection of organic acids is most commonly obtained using LC-MS and ^1^H-NMR spectroscopy platforms.

#### Vitamins

Vitamins are indispensable micronutrients, essential for biochemical reactions in all organisms. Humans are unable to synthesize most vitamins, hence, most of them need to be obtained exogenously, and some are produced by the gut microbiota (Stanton et al., 2005; Rossi and Amaretti, 2010).

Recently, Magnúsdóttir et al. (2015), using the PubSEED platform, assessed the genomes of 256 human gut bacteria involved in the biosynthesis of eight B-vitamins: biotin, folate, cobalamin, niacin, pantothenate, riboflavin, pyridoxine and thiamin. In particular, the authors demonstrated that each of the reported vitamins was produced by 40–65% of the 256 human gut microbes (Magnúsdóttir et al., 2015). Moreover, the absorption of some vitamins occurs in the small intestine after conjugation of vitamins with molecules (intrinsic factors) which are produced in the stomach. Since some vitamins are synthetized by the colonic microbiota, they are not adsorbed by the colon but are excreted in feces (Wilson, 2005).

Bifidobacteria strains have been recognized to be the strongest vitamin producers (Deguchi et al., 1985; Noda et al., 1994; Pompei et al., 2007), and in particular Bifidobacteria and Lactobacilli have been proposed as possible folate producers (Pompei et al., 2007; Kleerebezem and Vaughan, 2009). Folate (vitamin B9) is involved in various essential metabolic functions, such as DNA replication, repair and methylation, and synthesis of nucleotides, vitamins and certain AAs (LeBlanc et al., 2013). Folate is contained in leaf vegetables, cereals and liver.

The biosynthesis of thiamin (vitamin B1) consists of two pathways that unite in the final step of thiamin monophosphate production. Although thiamin diphosphate is the functional version of thiamin, all phyla (in particular Bacteroidetes and Fusobacteria), except Firmicutes are producers of thiamin monophosphate (Magnúsdóttir et al., 2015). Vitamin B1 is contained in pork meat, oatmeal, brown rice, vegetables, potatoes, liver, and eggs.

Biotin (vitamin B8) can be synthesized *de novo* from two pimeloyl precursors, namely malonyl-ACP and pimelate. Fusobacteria, Bacteroidetes and Proteobacteria synthetize biotin by different biochemical pathways, while Actinobacteria genomes lack the essential role of biotin biosynthesis (Magnúsdóttir et al., 2015). Vitamin B8 is contained in raw egg yolk, liver, peanuts and green leafy vegetables.

Riboflavin (vitamin B2) plays an essential role in cellular metabolism, being the precursor of the coenzymes flavin mononucleotide (FMN) and flavin adenine dinucleotide (FAD) (Li et al., 2014). Microbial riboflavin biosynthesis has been extensively described in *Bacillus subtilis* (Perkins and Pero, 2002) and *Escherichia coli* (Bacher et al., 1996, 2000). Vitamin B2 is contained in dairy products, bananas, popcorn, green beans and asparagus.

Cobalamin (vitamin B12) is the only vitamin that is exclusively produced by microorganisms, particularly by anaerobes (Roth et al., 1996; Martens et al., 2002; Smith et al., 2007).

Besides, the production of niacin and pyridoxine appears to be generated by Lactobacilli used in yogurt, cheese, and fermented foods (Shahani and Chandan, 1979; Alm, 1982).

Pantothenate (vitamin B5) is a coenzyme A (CoA) precursor and it can be synthesized *de novo* from 2-dihydropantoate and b-alanine. Bacteroidetes and several species of Proteobacteria and Actinobacteria have been demonstrated to be CoA producers (Magnúsdóttir et al., 2015). This vitamin is also contained in meat, broccoli, avocado. Detection of this vitamin is mostly obtained using LC-MS and ^1^H-NMR spectroscopy platforms.

Furthermore, Vitamin K operates as a co-factor for the enzymatic conversion of specific protein glutamyl to γ-carboxyglutamyl residues. The daily requirement of vitamin K is satisfied by dietary intake of phylloquinone and by the production of polyisoprenyl-containing compounds synthesized by the human gut microbiota (Suttie, 1995; Davidson et al., 1998; Martens et al., 2002). Green leafy vegetables such as spinach, egg yolks, and liver contain vitamin K.

### Bacterial-transformed compounds

#### Bile salts

The metabolism of bile salts is a well-known and basic skill of the gut microbiota metabolism, particularly associated to the genera of *Bacteroides, Clostridium, Lactobacillus, Bifidobacterium, Enterobacter, Eubacterium*, and *Escherichia* (Ridlon et al., 2006; Nicholson et al., 2012). Bacteria also contribute to the recovery of bile salts escaping from active transport in the distal ileum (Begley et al., 2006). The gut microbiota chemically modifies bile acids through a wide range of reactions, resulting in the formation of secondary and tertiary bile acids (Bortolini et al., 1997). Bile salts contribute to the absorption of dietary fats and lipid-soluble vitamins, facilitate lipid assimilation, maintain gut barrier function and regulate triglycerides, cholesterol and glucose by endocrine functions and energy homeostasis (Groh et al., 1993; Ridlon et al., 2006; Dawson et al., 2009).

However, bacterial bile salt hydrolysis has recently been considered as a risk factor for the development of colon cancer because it causes the formation of harmful secondary bile salts after an initial deconjugation step (De Boever et al., 2000). The secondary free bile acids can diffuse through the lipid bilayer of the membrane, thus being much more inhibitory for the cells than the conjugated forms (Mayo and van Sinderen, 2010). De Boever et al. (2000) have speculated a plausible mechanism for the protective properties of probiotic *Lactobacillus reuteri*, which could precipitate the deconjugated bile salts by a physical binding, making the harmful bile salts less bioavailable. Bile salts have antimicrobial activity on gut microbes with inhibitory effects on Bacteroidetes and Actinobacteria microbial population (Islam et al., 2011), but high levels of these biomarkers in serum and urine are correlated with liver diseases (Bathena et al., 2015). The LC-MS and ^1^H-NMR spectroscopy platforms are the main techniques used to detect bile acids.

#### Polyphenols

Polyphenols are considerably bioactive components in the diet (Manach et al., 2004). Hydroxycinnamic acids and flavonoids are the two major classes of polyphenols. Fruits commonly contain caffeic acid, representing the most abundant hydroxycinnamic acid (Clifford, 2004). In particular, the chemically derived chlorogenic acid is commonly present in apples, berries and kiwifruit, in vegetables such as potatoes (Manach et al., 2004) and, in high concentrations, in coffee (Clifford, 2004).

Recent studies have demonstrated that gut bacteria, including strains of *Lactobacillus* and *Bifidobacterium*, can metabolize chlorogenic acid to form caffeic acid and quinic acid (Couteau et al., 2001; Taverniti and Guglielmetti, 2012; Amaretti et al., 2015; Marín et al., 2015; Raimondi et al., 2015), while caffeic acid is further metabolized to form the μ-coumaric acid (3-hydroxycinnamic acid), 3-hydroxylphenylacetic acid and dihydroxyphenylpropionic acid (Konishi and Kobayashi, 2004). The 3,4- dihydroxyphenylacetic acid also derives from the colonic catabolism of rutin (Jaganath et al., 2009). Conversely, phenolic acid metabolites of rutin are not produced in germ-free mice, implying that ring-fission products are generated only by intestinal bacteria (Selma et al., 2009; Parkar et al., 2013). The polyphenol detection is performed using LC-MS, GC-MS, and ^1^H-NMR spectroscopy platforms.

#### Lipids

Significant amounts of glycerol derive from daily dietary intake and/or from *in situ* microbial production, or from enterocyte desquamation. Some gut bacteria may anaerobically reduce glycerol to 1,3-propanediol, with the production of the intermediate 3-hydroxypropanal. The accumulation of this metabolite leads to the formation of reuterin, which is known for its antimicrobial properties (De Weirdt et al., 2010). Lipids are also involved in intestinal permeability, in the regulation of glucose homeostasis *via* intestine-brain-liver-neural axis, in the promotion of chronic systemic inflammation by LPS, in the improvement of hyperinsulinemia by conjugated fatty acids (FAs), in the enhancement of the immune system, and in the alteration of lipoprotein profiles. *Bifidobacterium, Roseburia, Lactobacillus, Klebsiella, Enterobacter, Citrobacter, Clostridium* genera have been recognized as the main actors in lipid metabolism (Nicholson et al., 2012). The platform constituted of LC-MS, GC-MS, and ^1^H-NMR spectroscopy is mainly used to perform lipid detection.

#### Amino acids

The bacterial fermentation of proteins, occurring in the distal colon, leads to AAs fermentation products having some relevance for health (Macfarlane and Macfarlane, 1995). For instance, AAs deamination produces ammonia, whereas decarboxylation produces amines, which may have toxicological effects (Silla Santos, 1996). High ammonia concentrations have been found to act as tumor promoters (Clinton et al., 1988). Bacterial degradation of AAs cysteine and methionine leads to the formation of H_2_S, which is toxic and has also been reported to be responsible for inhibition of butyrate oxidation in colonocytes (Roediger et al., 1993). Furthermore, the anaerobic fermentation of the aromatic AAs tyrosine and tryptophan by colonic bacteria produces phenols and indoles respectively, which are eventually excreted in the urine (Macfarlane and Macfarlane, 1997). Phenols, such as *p*-cresol, have been proposed to act as procarcinogens in colon cancer (Bone et al., 1976). Interestingly, gut bacterial production of *p*-cresol is significantly related to autism (Clayton, 2012; De Angelis et al., 2013), and *C. difficile* appears to be a significant *p*-cresol producer (Sivsammye and Sims, 1990).

Finally, certain species of *Clostridium* (Moss et al., 1970) and *Peptostreptococcus anaerobius* (Lambert and Moss, 1980) can convert phenylalanine to benzoic acid in a multistep process with phenylpropionic acid (toxic metabolic product) acting as intermediate (Macfarlane and Macfarlane, 1997; Smith and Macfarlane, 1997). The AAs are mainly detected using LC-MS and ^1^H-NMR spectroscopy techniques, and also using FT-ICR-MS.

## Biological action of the gut microbiota in healthy and diseased subjects

In recent years, growing attention has been targeted to the role of the gut microbiota in the pathogenesis of gastrointestinal (GI) diseases (Lozupone et al., 2012). The alteration of the interplay between host and microbes at the gut level stimulates perturbation of the homeostasis and leads to the development of disorders.

The microbial ecosystem undergoes changes when the equilibrium is broken, which leads to the modification of the bacterial metabolic activity, and/or to transfers in the distribution of local bacterial communities. In fact, the phylotype complexity regulates the equilibrium between pathogenic and commensal taxa at the GI interface (Prakash et al., 2011).

The intestinal gut dysbiosis is associated with a plethora of children and adult diseases, including genetic (i.e., cystic fibrosis [CF]), inflammatory (i.e., inflammatory bowel diseases and syndrome [IBDs, IBS], Chron's [CD], ulcerative colitis [UC], and celiac disease), metabolic (i.e., diabetes, obesity and non-alcoholic fatty liver disease [NAFLD]), and allergic (i.e., atopic dermatitis, food allergies) disorders (Del Chierico et al., 2012), and neuropathologies (i.e., autism) (Figure 2).

Indeed, metabolomics is an approach allowing to perform a careful diagnosis of diseases, since metabolite profiles have a high resolution power, which enable to separate the groups based on microbial community profiles (Dicksved et al., 2008). Moreover, metabolites represent the terminal enzymatic process signature occurring in the gut, and the molecules within the pathways range allow to distinguish healthy from diseased subjects, as well as among disease phenotypes (Jansson et al., 2009; Table 3).

**Table 3:** **Correlated microbes and metabolites to diseases and the relative metabolomic platforms**.

**Disease**	**Biofluids**	**Correlated microbes**	**Correlated metabolites**	**Platforms**	**References**
IBD/IBS	Feces Urine	Actinobacteria, Firmicutes (*Faecalibacterium prausnitzii, Clostridium* clusters XIVa and IV), Proteobacteria *(Escherichia coli*)	IBS: hydrogen and esters; IBD: alcohols, esters, indoles, phenols, acetone, sulfur compounds, propanoic and butanoic acids, phenol and *p*-cresol, hippurate; CD: tyrosine, dopamine, tryptophan, phenylalanine, isoleucine, leucine, lysisne, bile acid (i.e., glycocholate); UC: cadaverine and taurine	SPME-GC-MS; Breath gas analyzer; ^1^H-NMR; FT-ICR-MS	Best and Laposata, 2003; Marchesi et al., 2007; Jansson et al., 2009; Kumar et al., 2010; Ahmed et al., 2013; Stephens et al., 2013; Walton et al., 2013; Bjerrum et al., 2015; De Preter, 2015; De Preter et al., 2015
Obesity	Urine Serum	Firmicutes (*Clostridium* spp), Proteobacteria, Bacteroidetes, *Bifidobacterium* spp.	hippurate, 4-hydroxylphenylacetic acid, phenylacetylglycine, FFA, BCAA, primary bile acids (i.e., cholic, chenodeoxycholic acid), secondary bile acids (i.e., lithocholic acid)	^1^H-NMR; LC-ESI-Q-TOF	Veselkov et al., 2009; Respondek et al., 2013; Zhang et al., 2015; Paul et al., 2016
Cystic fibrosis (CF)	Breath condensate	*Pseudomonas aeruginosa, Clostridium* clusters *XIVa and IV, Clostridium acetobutylicum, F. prausnitzii, Eubacterium limnosum, Eubacterium biforme, E coli, Bifodobacterium* spp.	C5–C16 hydrocarbons and N-methyl-2-methylpropylamine ethanol, methanol, acetate, 2-propanol, lactate, dimethyl sulfide and acetone	^1^H-NMR; GC-TOF-MS	Wang et al., 2006; Robroeks et al., 2010; Montuschi et al., 2012; Scalan et al., 2012; Schippa et al., 2013
Non-alcoholic Fatty Liver Disease (NAFLD)	Feces	*Oscillospira*, Rickenellaceae, *Parabacteroides, Bacteroides fragilis, Sutterella*, Lachanospiraceae	ethanol, esters (i.e., ethyl propionate, methyl pentanoate, methyl acetate), 4-Methyl-2-pentanone, 1-butanol and 2-butanoate	SPME-GC-MS	Raman et al., 2013; Del Chierico et al., 2016
Celiac Disease	Feces Serum Urine	*Lactobacillus, Enterococcus*, Bifidobacteria, *Bacteroides, Staphylococcus, Salmonella, Shighella, Klebsiella*	acetoacetate, glucose and 3-hydroxybutyric acid, indoxyl sulfate, meta-[hydroxyphenyl] propionic acid, and phenylacetylglycine, 1-octen-3-ol, ethanol and 1-propanol, AAs (i.e., proline, methionine, histidine, and tryptophan, isoleucine, asparagine, valine, creatinine), choline, lactate, methylamine, non-anal, 4-Methyl-2-hexanone, ethyl acetate and pyruvate	Breath gas analyzer; GC-TOF-MS; NMR	Cani et al., 2008; Di Cagno et al., 2011; Calabrò et al., 2014; Francavilla et al., 2014
Food allergies	Feces	Bifidobacteria, Lactic Acid Bacteria (LAB), Bacteroides, Clostridia	SCFAs (i.e., butyric and acetic acid), lactic acid and threonine	GC-MS; NMR	Francavilla et al., 2012
Neuropathology	Feces Serum	*Faecalibacterium, Ruminococcus, Clostridium*, Lachnospiraceae, Eubacteriaceae, Bacteroidetes, *Alistipes, Akkermansia*, Sutterellaceae, Enterobacteriaceae, *Bifidobacterium*	tryptophan–nicotinic acid, sulfur metabolic pathways, indolepyruvate, *p*-cresol and ethyl sulfate	LC-MS; GC-MS; SPME-GC-MS	Kidd, 2002; Oresic et al., 2008; De Angelis et al., 2013; Hsiao et al., 2013

### Inflammatory bowel disease/inflammatory bowel syndrome (IBD/IBS)

As concerns inflammatory diseases at GI tract, it is well known that the microbiota results to be abnormal both in IBD and IBS, showing decreased levels of Actinobacteria and Firmicutes, and high levels of Proteobacteria compared to healthy subjects (Kinross et al., 2011; Carroll et al., 2012; Mukhopadhya et al., 2012; De Preter et al., 2013).

Irregular microbial fermentation leads to a high production of hydrogen (in IBS), indole, phenols and others (Kumar et al., 2010). In fact, bacteria release volatile organic compounds (VOCs), determined by SPME-GC-MS, as by-products of metabolism. Hence, the rising acceptance of the gut microbiota involvement in the pathogenesis of IBD has led to the use of fecal matrix as a sample to determine metabolite profiling (Walton et al., 2013). Indeed, specific microbial VOCs profiles can provide specific biomarker candidates for diagnostic purposes (Schöller et al., 1997; Lechner and Rieder, 2007; Bunge et al., 2008).

Walton et al. (2013) observed differences among patient categories (IBD, UC, and CD) based on compounds detected in fecal samples, such as SCFAs and their corresponding alcohols, esters, and molecules, such as indoles and phenols, acetone and sulfur compounds.

The concentrations of propanoic and butanoic acids, revealed by using GC-MS, represent a source of energy affecting colonic mucosal growth, and these concentrations were found to be higher in CD subjects, compared to healthy controls (Best and Laposata, 2003), while acids such as oleic, stearic, palmitic, linoleic and arachidonic were higher in the ileum of CD patients (Jansson et al., 2009). Ahmed et al. observed an increase of esters in diarrhea predominant IBS patients using the SPME-GC-MS technique (Ahmed et al., 2013). On the other side, Walton et al. (2013) detected high levels of indole, phenol and *p*-cresol, generally considered to be toxic for the gut, in CD and UC groups compared to controls.

Moreover, Jansson et al. (2009), using FT-ICR-MS, detected several masses related to metabolites within the tyrosine metabolic pathway, which differentiated CD from healthy controls. In particular, dopaquinone (a dopa oxidation product and intermediate in the melanin formation from tyrosine) was significantly elevated in CD patients compared to healthy subjects. The authors also indicated that tryptophan and phenyalanine were related to the ileum CD phenotype. It was also observed, using ^1^H-NMR spectroscopy, that AAs in CD patients with active disease showed a different profile (i.e., alanine, isoleucine, leucine, and lysine) compared to CD patients in remission (Marchesi et al., 2007). Furthermore, metabolites related to bile acids pathways (i.e., glycocholate) were found in CD patients in remission (Jansson et al., 2009).

Other studies (Marchesi et al., 2007; Bjerrum et al., 2015; De Preter, 2015; De Preter et al., 2015) using ^1^H-NMR and GC-MS showed a depletion of bacterial products, such as SCFAs, branched chain FAs, dimethylamine and trimethylamine, and high levels of AAs, suggesting a breakdown of the normal bacterial ecology that induces dysbiosis, as a cause or a consequence of the disease in IBD. Le Gall et al. (2011), using Denaturing Gradient Gel Electrophoresis (DGGE), discriminated UC patients from controls, and also found a correlation between the gut microbiota composition and the metabolite composition (high levels of cadaverine and taurine). Besides, UC patients with high levels of AAs showed low fecal concentrations of *Faecalibacterium prausnitzii* and therefore a small amount of butyric acid, since this bacteria is an SCFA producer (De Preter et al., 2015).

The correction of some microbial fermentations by antibiotics or diet could improve symptoms and abnormal fermentation, which, in some IBS cases, is believed to underlie food intolerances (Nanda et al., 1989). Furthermore, using NMR spectroscopy analysis of urine, it is possible to discriminate IBD patients from controls based on metabolite content of hippurate (Stephens et al., 2013). The levels of hippurate were found to be lower in IBD patients compared to controls, suggesting that hippurate is a biomarker of IBD. In particular, hippurate or N-benzoylglycine is a mammalian-microbial co-metabolite deriving from the microbial fermentation of dietary aromatic compounds (polyphenols, purines, or aromatic AAs) to benzoic acid, further conjugated to glycine in the liver (Williams et al., 2010).

### Obesity

There is evidence that obese and healthy children (HC) show a different gut microbiota profile. Several mechanisms are involved in the energy metabolism regulation and represent the link between the gut microbiota and the metabolic disease pathophysiology, such as energy harvesting from the diet, fat storage regulation, and energy homeostasis of peptide synthesis (Cani and Delzenne, 2009; Krajmalnik-Brown et al., 2012).

In a study on mouse models, Zhang et al. (2011), using LC-ESI-Q-TOF/NMR, found in urine increased excretion of hippurate, 4-hydroxylphenylacetic acid and phenylacetylglycine, and decreased excretion of acetate and lactate, related to body weight gain and to an alteration of gut microbial changes (Veselkov et al., 2009). Moreover, Paul et al. (2016) identified, with the support of ^1^H-NMR metabolomics, a maternal metabolic signature that may be related to programming offspring obesity risk in rats. In particular, pregnant rats showed high levels of circulating ketone bodies and free FA (FFA), especially associated with gestational diabetes (Catalano, 2010). The branched chains AA (BCAA) have also found to be related to increased insulin resistance (Scholtens et al., 2014). In humans, FFA are transferred through the placenta from mother to child and are used for lipogenesis. FFA, together with the circulating ketons, may play an important role in the early deposition of excess of body fats in offspring. The production of these molecules in serum metabolome is normalized when it is associated to diet enriched with oligrofructosaccharides (FOS) and to gut microbiota modulation (Paul et al., 2016). Respondek et al. (2013) studied the effects of FOS on the composition of the fecal microbiota and the metabolic parameters in animal models of diet-induced obesity (Respondek et al., 2013). The authors firstly found that the strains particularly stimulated by FOS were *Clostridium coccoides, Ruminococcus torques*, and *Dorea longicatena*, and the fecal metabolites modulated by the supplementation, analyzed by using LC-ESI-TOF-MS, were primary bile acids (i.e., cholic and chenodeoxycholic acid) and secondary bile acids (i.e., lithocholic acid).

### Cystic fibrosis (CF)

Regarding CF disease, the majority of studies on the microbiota metabolome concern lung and the upper airways. It was observed that in CF the production of isoprene is associated to either Gram+ and Gram- species (Kuzma et al., 1995), while the production of hydrogen cyanide is prevalently associated to *Pseudomonas aeruginosa*, (Carterson et al., 2004; Cody et al., 2009). Robroeks et al. (2010) analyzed exhaled breath samples by GC-TOF-MS, and they managed to discriminate between CF and healthy controls, mainly based on the presence of C5–C16 hydrocarbons and N-methyl-2-methylpropylamine. Barker et al. (2006) reported a significantly lower level of dimethyl sulfide (probably associated to microbial metabolism), detected by GC-MS in CF patients compared to controls. On the other side, Montuschi et al. (2012), using ^1^H-NMR forexhaled breath condensate (EBC) analysis, detected significantly higher values of ethanol, acetate, 2-propanol and acetone in CF patients, which differentiated them from controls, whereas acetate, ethanol, 2-propanol and methanol were found to be relevant metabolites for distinguishing between patients with stable CF and patients with unstable CF. Moreover, 2-Propanol, which represents an enzyme-mediated product of acetone reduction, was detected in a breath sample of CF colonized by *P. aeruginosa* (Wang et al., 2006). The high level of ethanol in EBC samples of CF, detected by MS based metabolomics, could also be related to the decreased capacity of *P. aeruginosa* to oxidize ethanol to acetate (Wang et al., 2006). On the contrary, elevated acetate concentrations in healthy subjects may reflect resident bacteria in the oral cavity, such as *Streptococcus mutans*, debasing pyruvate into metabolic end products, such as acetate and lactate (Korithoski et al., 2008).

### Non-alcoholic fatty liver disease (NAFLD)

Several VOCs, including ethanol, seem to be produced by colonic bacteria and may have toxic effects on the host after intestinal absorption and delivery to the liver *via* the portal vein (Raman et al., 2013). Moreover, Raman et al. (2013), using SPME-GC-MS, identified esters (i.e., ethyl propanoate, butyl butanoate, methyl pentanoate, methyl acetate) in fecal samples of obese NAFLD patients more frequently than in healthy controls.

The bacterial production of SCFAs and ethanol by several gut microbes is well known, but very little is known about bacteria and biochemical pathways that may be involved in ester production in the intestinal microbiota, even though most of the esters linked to NAFLD are derivatives of short chain aliphatic alcohols and carboxylic acids (Raman et al., 2013). Also Del Chierico et al. (2016) evaluated the gut microbiota profiling of NAFLD and obese patients. The authors evidenced, with the support of multivariate analysis, that OTUs such as *Oscillospira*, Ricknellaceae, *Parabacteroides, Bacteroides fragilis, Sutterella*, and Lachnospiraceae, and metabolites such as 4-Methyl-2-pentanone, 1-butanol and 2-butanone (detected with SPME-GC-MS), discriminated NAFLD from healthy subjects (Del Chierico et al., 2016).

### Celiac disease

As regards studies on celiac disease, it was clearly shown that metabolic differences between controls and celiac patients exist (Calabrò et al., 2014).

The main differences detected coupling MS-and NMR- based metabolomics approaches in celiac patients compared to controls were lower levels of several AAs, as asparagine, isoleucine, methionine, proline, and valine, and also methylamine, pyruvate, creatinine, choline, methyl glutarate, lactate, lipids, and glycoproteins, and higher levels of glucose and 3-hydroxybutyric acid in serum and acetoacetate in the urine of celiac patients (Calabrò et al., 2014). The same authors also found higher levels of some metabolites related to the gut microbiota in the urine, such as: indoxyl sulfate, meta-[hydroxyphenyl] propionic acid (m-HPPA), and phenylacetylglycine. In fact, M-HPPA in urine mostly originates from the gut microbiota, being one of the many products of the microbial mediated breakdown of plant phenolic compounds, such as caffeic acid and its conjugate chlorogenic acids (Phipps et al., 1998). Besides, Di Cagno et al. (2011) analyzed, using SPME-GC-MS and ^1^H-NMR, VOCs, and AAs of fecal and urine samples of treated (gluten free diet) celiac children. The samples showed higher levels of free AAs (proline, methionine, histidine, and tryptophan) and lower levels of SCFAs, as butyric, isocaproic, and propanoic acids compared to controls. In this study, it was also found that the levels of some alcohols, such as 1-octen-3-ol, ethanol and 1-propanol were higher in treated celiac children compared to controls and it was hypothesized that when alcohol production is correlated with intestinal bacteria synthesis this may also induce endotoxemia (Cani et al., 2008).

In another study, saliva samples have been analyzed, using SPME-GC-MS, revealing high levels of non-anal, 4-methyl-2-hexanone, and ethyl-acetate in treated celiac children (Francavilla et al., 2014). These findings suggest the presence of microbial metabolic activities at the oral cavity level (by Firmicutes, Actinobacteria and Bacteroidetes) that may also affect the synthesis of VOCs (Kusano et al., 2013).

### Food allergies

Finally, the gut microbiota is believed to be associated with food allergies. In particular, the prevalence of atopic diseases, including eczema and asthma, suggests that the modulation of the immune response mechanisms in the gut can directly affect the development of allergic diseases and the development of tolerance (Watanabe et al., 2003; Penders et al., 2007). Moreover, the advent of dysbiosis during the early post-natal period may further pre-dispose individuals to later inflammatory, immune, and allergic disorders (Francavilla et al., 2012).

However, there is still little scientific evidence on the relation between the gut microbiota metabolome and food allergy. A study by Francavilla et al. (2012) describing the metabolome of infants with cow's milk allergy, was conducted using the combinate of SPME-GC-MS and ^1^H-NMR techniques on a group of children fed with hydrolyzed formula with no lactose (CMA-NL), and a group of children fed with lactose-containing (CMA-L) formula compared with controls. The authors found that the addition of lactose to the formula resulted in a significant increase of Bifidobacteria and LAB counts, and a decrease of Bacteroides/Clostridia. Consequently, the levels of SCFAs increased, especially for acetic and butyric acids, in controls and CMA-L compared to CMA-NL infants. The same trend was found for lactic acid and threonine.

### Neuropathology

The composition of the intestinal microbiota plays a key role in neuro-gastroenterology, which deals with the interactions between the central nervous system and the gut (gut–brain axis). Numerous neuropathological diseases, such as autism spectrum disorder (ASD), are probably associated with the gut microbiota and thus the possibility to influence this connection is alluring (Holmes et al., 2011), even if to date there are still few studies investigating this field. De Angelis et al. (2013) used SPME-GC-MS and ^1^H-NMR to study the fecal microbiota and the metabolome of children with Pervasive Developmental Disorder Not Otherwise Specified (PDD-NOS), and children with ASD compared to healthy controls. The authors found an altered composition of the microbiota and VOCs, which were partially different between children with PDD-NOS and ASD. The main biological significance of this work was related to the increased levels of *Clostridum* in PDD-NOS and ASD and the decreased levels of some health promoting bacteria (i.e., *Bifidobacterium*) and metabolites, such as free AA and SCFAs in PDD-NOS, in ASD children compared to controls (De Angelis et al., 2013). Furthermore, Kidd (2002) found that subjects with ASD together with their non-ASD siblings, presented with a deep alteration in the tryptophan–nicotinic acid and sulfur metabolic pathways (Kidd, 2002; Oresic et al., 2008). Important metabolic phenotype differences were observed between ASD and controls with perturbations in the relative patterns of urinary metabolites related to the gut microbiota (Kidd, 2002).

Hsiao et al. (2013) used GC- and LC-MS platforms to study the oral treatment with human commensal *Bacteroides fragilis* (that corrects gut permeability) to modulate the microbial composition and the related defects in communicative behaviors in mouse models with maternal immune activation (MIA), showing GI barrier defects and microbiota alterations in displaying features of ASD. The authors detected the presence of indolepyruvate 4-ethylphenylsulfate and *p*-cresol in mice serum metabolome, presumably deriving from microbial metabolism. Furthermore, since *B. fragilis* improves intestinal health, it could also have a role in regulating intestinal permeability and metabolic homeostasis (Nicholson et al., 2012).

## Conclusions and perspective

The challenge of systems medicine is to interpret the body structure as a whole system and not as a sum of single parts (Moco et al., 2013). To pursue this aim, the wide range of top-down systems biology analyses should be used to interpret the metabolic interactions between the host and its gut microbiota, and to comprehend how these interactions affect the physiological and pathological conditions. Furthermore, the combination of these techniques with genome analysis may lead to a holistic view of the metabolic pathways, which can also be backed up using mathematical models and statistical assessment of data. In fact, by managing data it is possible to achieve a higher level of biological understanding. Therefore, novel algorithms and statistical analysis need to be improved to integrate the “omics” data, and a stochastic model of metabolic networks needs to be introduced to lead to a novel knowledge of co-regulation in biochemical networks.

The metabolomics approach may identify physiological and clinical biomarkers that are not obtainable using targeted methods (Weckwerth and Morgenthal, 2005).

In conclusion, the generation of new gut microbiota biomarkers will offer the chance to associate complex metabolic pathways with the etiology of different diseases, in order to evaluate the causal relationship between metabolites and pathogenesis. Moreover, these novel biomarkers could lead to the development of mechanistic hypotheses that could be targeted to the development of nutritional and personalized therapy tools in early disease prediction in asymptomatic conditions, and enable a more accurate prognosis of the disease progress.

## Author contributions

PV, conceived and wrote the manuscript. FD, participated in the writing and produced tables and figures. LP, supervised and reviewed the manuscript.

### Conflict of interest statement

The authors declare that the research was conducted in the absence of any commercial or financial relationships that could be construed as a potential conflict of interest.
